# Phase-Specific Resveratrol Supplementation in Distraction Osteogenesis: Supporting the Redox Environment of Bone Healing

**DOI:** 10.7759/cureus.98530

**Published:** 2025-12-05

**Authors:** Androniki Drakou, Angelos Kaspiris, Markos Psifis, Elias Vasiliadis, Anna Lenti, Sophia Chatziioannou, Spiridon Pnevmaticos

**Affiliations:** 1 Department of Orthopedics, Laiko General Hospital of Athens, Athens, GRC; 2 Third Department of Orthopedics, National and Kapodistrian University of Athens, KAT Hospital, Athens, GRC; 3 Department of Orthopedics, Venizeleio General Hospital, Heraklion, GRC; 4 Third Department of Orthopedics, National and Kapodistrian University of Athens, KAT Trauma Hospital, Athens, GRC; 5 Department of Orthopedics and Traumatology, Laiko General Hospital of Athens, Athens, GRC; 6 Second Department of Radiology, National and Kapodistrian University of Athens, Athens, GRC; 7 Third Department of Orthopedics, KAT General Hospital, Athens, GRC

**Keywords:** distraction osteogenesis (do), oxidative dna damage, oxidative stress, redox status, resveratrol

## Abstract

Introduction: This experimental study investigates the correlation between resveratrol administration during the endochondral ossification process of distraction osteogenesis (DO) for the treatment of bone defects and the rate of systemic and local oxidative stress in a rabbit model of femoral DO.

Materials and methods: Twenty New Zealand white rabbits underwent femoral osteotomy and external fixation. The experimental group (n=9) received oral resveratrol (10 mg/kg/day) during the distraction phase (days 10-20), while controls (n = 9) received no antioxidant. Blood and regenerated tissues were analyzed for oxidative stress markers (8‑hydroxy‑2’-deoxyguanosine {8‑OHdG}, total antioxidant capacity {TAC}, and glutathione {GSH}) at multiple time points. Histological and immunohistochemical analyses were performed at the endpoint.

Results: Resveratrol significantly reduced 8-OHdG levels and preserved TAC and GSH concentrations during the distraction phase (p<0.05). Tissue staining revealed decreased oxidative DNA damage in the regenerate zone of treated animals. No significant radiographic differences in regenerate density were observed at day 49.

Conclusions: Resveratrol administration during distraction mitigated oxidative stress both systemically and locally, suggesting a cytoprotective effect on bone-regenerating cells. Although radiological differences were not apparent within the study period, the redox-modulating properties of resveratrol may support more favorable regenerative outcomes over time. These findings justify further investigation into phase-specific antioxidant protocols in DO, including human application.

## Introduction

Distraction osteogenesis (DO) is a well-established surgical technique used to address critical-sized bone defects and limb length discrepancies [1]. The process involves a latency phase, during which an early fibrocartilaginous callus forms, followed by a distraction phase where gradual mechanical tension induces new bone formation within the distraction gap, and finally a consolidation phase that allows maturation and remodeling of the regenerate bone [1]. Although highly effective, DO is associated with a prolonged treatment time and the potential for complications related to incomplete regenerate formation or delayed mineralization [1].

Oxidative stress has been shown to negatively affect bone regeneration by disrupting the balance between osteoblast-mediated bone formation and osteoclast activity, as well as by impairing the mineralization capacity of chondrocytes and osteoblasts [2]. Reactive oxygen species (ROS) accumulation can lead to lipid peroxidation, protein oxidation, and DNA damage, ultimately interfering with the complex cellular and molecular processes required for successful DO [3]. Antioxidant strategies aimed at mitigating oxidative stress may therefore represent a promising adjunct to enhance the quality and speed of regenerate formation.

Resveratrol, a naturally occurring polyphenolic compound found in grapes and other plants, has gained significant attention for its potent antioxidant, anti-inflammatory, and bone-protective properties [4-6]. In preclinical models, resveratrol has been shown to promote osteoblast differentiation, enhance mineralization, and reduce oxidative stress-induced cellular damage [7]. Importantly, the existing evidence supports that resveratrol can drive hypertrophy in chondrocytes (which is a precursor to mineralization), supports chondrogenic extracellular matrix (ECM), and enhances mineralization in osteoblastic lineages [7-9]. However, as far as the existing literature indicates, resveratrol has not been directly tested in a distraction osteogenesis model focusing on the latency period and the mineralization of chondrocytes.

In this study, we aimed to evaluate the effects of resveratrol administration on regenerate formation during distraction osteogenesis in a rabbit femoral osteotomy model. To maximize its potential impact on mineralization and ossification, resveratrol was administered exclusively during the distraction phase (days 10-20), a period during which endochondral and intramembranous ossification mechanisms are highly active. We hypothesized that targeted antioxidant therapy with resveratrol during this critical window would improve regenerate quality, reduce oxidative stress, and enhance the overall biological response to DO.

## Materials and methods

Study design

Twenty adult male New Zealand white rabbits (mean weight: 1,800 g; range: 1,500-2,000 g) were included in this experimental study. All animals were housed individually in standard cages under controlled environmental conditions (temperature 20°-22°C; 12 h light/dark cycle) with ad libitum access to water and standard laboratory chow.

The rabbits were randomly allocated into two groups (n=10/group). The first constituted the control group that received no intervention other than the surgical procedure, while the second was the experimental group in which the animals received oral resveratrol (10 mg/kg/day) during the distraction phase (days 10-20). Two animals (one from each group) died suddenly during the experimental period; therefore, the final sample size was nine rabbits per group. All procedures were approved by the Institutional Animal Care and Use Committee and conducted at the Institutional Experimental Animal Center of Biomedical Research Foundation of the Academy of Athens (BRFAA).

Surgical procedure

On day one, rabbits were anesthetized with xylazine (0.2 mg/kg) and ketamine hydrochloride (20 mg/kg) administered intramuscularly. Infection prophylaxis with cefazolin sodium (20 mg/kg/day) was administered preoperatively and for two days postoperatively.

**Figure 1:**
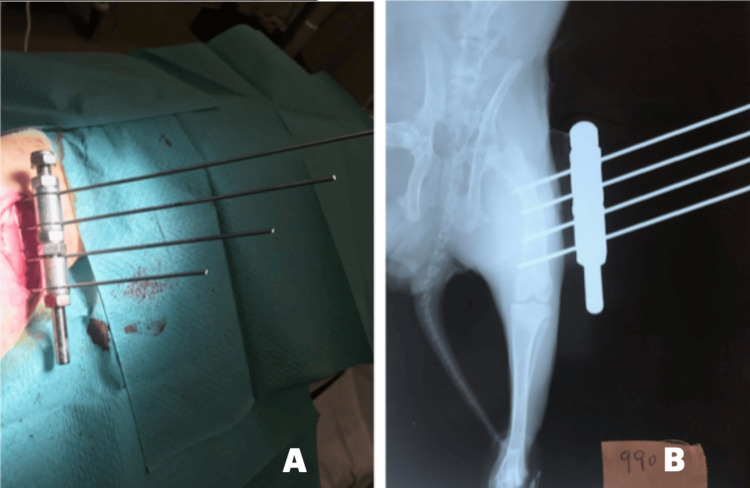
External fixation and post-operative radiographic assessment. (A) Intraoperative photograph showing the external fixator applied to the right femur of the rabbit. (B) Post-operative posteroanterior radiograph of the right femur demonstrating the position and alignment of the fixator and underlying bone.

A longitudinal lateral incision was made over the mid-diaphysis of the right femur, and the periosteum was carefully elevated. A transverse mid-diaphyseal osteotomy was performed, and the bone was stabilized using a monolateral external fixator (Orthofix-type) (Figure 1, panel A).

The periosteum and soft tissues were sutured in layers. Postoperatively, the animals were allowed free cage activity. The follow-up was categorized into three periods. Specifically, five days post-osteotomy composed the latency period, while 10-20 days (0.25 mm every 12h; total 0.5 mm/day) and 20 days post-distraction constituted the distraction and consolidation phases, respectively.

Radiological evaluation

Standard posteroanterior radiographs of the operated femur were performed immediately after the operative intervention and on the 49th day, which was the day before euthanasia (Figure 1, panel B). All the radiographs were independently reviewed by two blind observers using a standard scoring system to evaluate regenerate formation, corticalization, and bridging callus. No statistically significant differences in radiological appearance were observed between the control and experimental groups.

Blood sampling and oxidative stress marker analysis

Serial venous blood samples (2 mL) were collected from the marginal ear vein under minimal restraint at the following time points: prior to osteotomy (T0, baseline); on the fifth day (T1, latency); on the 10th, 15th, and 20th days (T2, distraction); and on the 30th and 50th days (T3, consolidation). The plasma was separated by centrifugation (3,000 g, 10 min, 4°C) and stored at -80°C until analysis.

In order to evaluate the rate of oxidative stress, antioxidant markers such as 8-hydroxy-2’-deoxyguanosine (8-OHdG), total antioxidant capacity (TAC), and glutathione (GSH) were measured using an enzyme-linked immunosorbent assay (ELISA)-based assay with the F-12 clone of mouse antibody (8-OHdG {F-12}: sc-393870; Dallas, TX: Santa Cruz Biotechnology, Inc.), a colorimetric assay (Ann Arbor, MI: Cayman Chemical), and an enzymatic recycling assay (Ann Arbor, MI: Cayman Chemical), respectively. All measurements were normalized to plasma protein levels.

Tissue collection and immunohistochemistry

**Figure 2:**
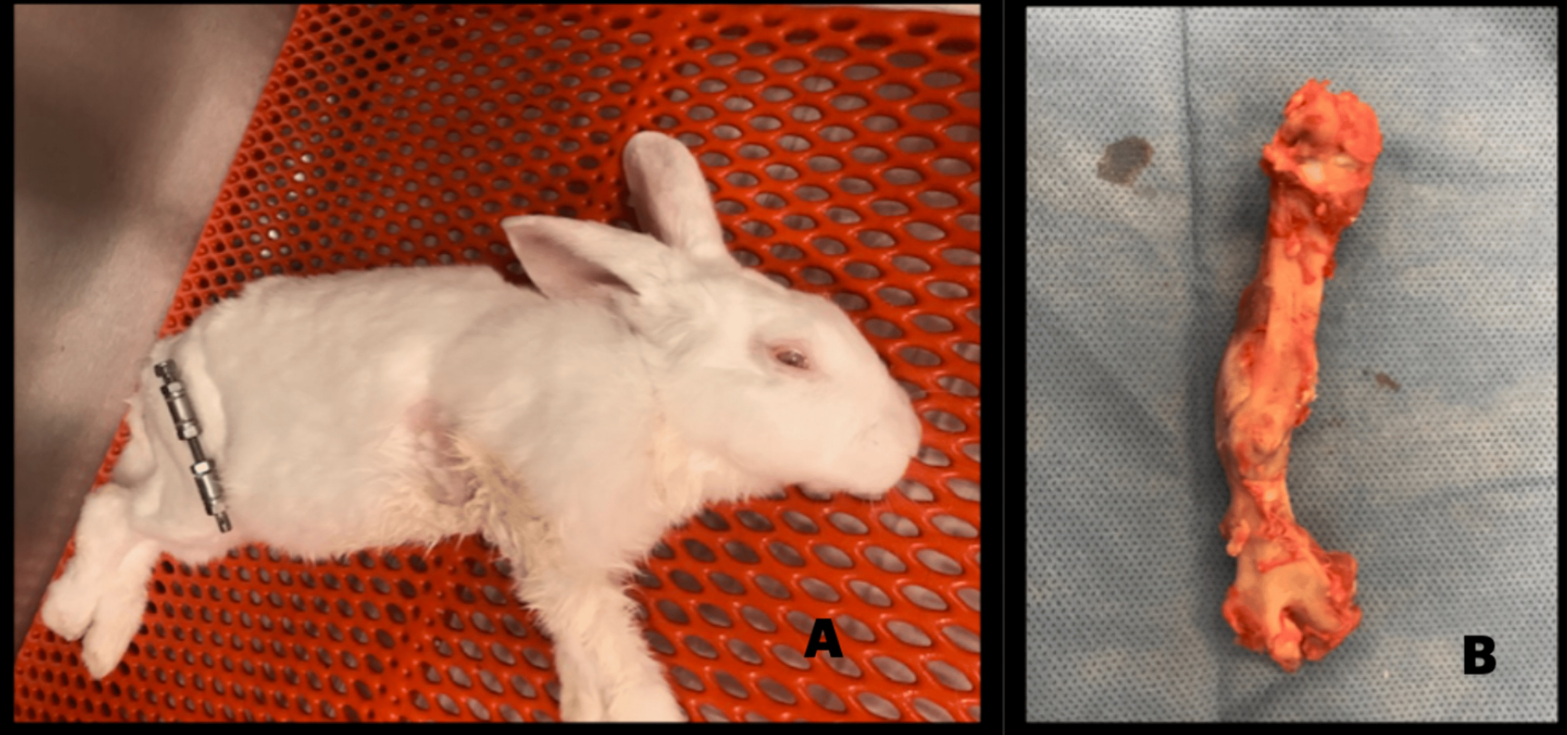
Postmortem femur resection. The images show (A) a postmortem photograph of the animal with the femoral fixator in situ and (B) postmortem femur resection.

On day 50, animals were euthanized by intravenous pentobarbital overdose (150 mg/kg) (Figure 2, panel A). The operated femurs, including regenerated bone, were carefully harvested (Figure 2, panel B). Two types of samples were collected. Notably, the radiologically confirmed regenerate zone was fixed for histology and immunohistochemistry, whereas the adjacent tissue samples were snap-frozen in liquid nitrogen and stored at -80°C for biochemical analysis (TAC and GSH).

Specimens were fixed in 10% neutral-buffered formalin for 48 h, decalcified in 10% ethylenediaminetetraacetic acid (EDTA) (pH 7.4) for two to three weeks, dehydrated, and paraffin-embedded. Serial 4-5 µm sections were cut and mounted on poly-L-lysine-coated slides. The 8-OHdG (F-12) mouse monoclonal antibody (Dallas, TX: Santa Cruz Biotechnology, Inc.) was used to evaluate the tissue distribution and cellular localization of 8-OHdG. Immunohistochemical staining was performed on paraffin-embedded sections of fracture callus. Tissue sections (5 μm thick) were deparaffinized in xylene, rehydrated through graded ethanol, and subjected to antigen retrieval using citrate buffer (pH 6.0) in a microwave oven for 20 minutes.

Endogenous peroxidase activity was quenched using 3% hydrogen peroxide for 10 minutes. Non-specific binding was blocked with 5% normal serum for 30 minutes at room temperature. The primary antibody 8-OHdG (F-12) was applied at a starting dilution of 1:50, and further tested within a dilution range of 1:50 to 1:500 to determine optimal staining intensity and specificity.

**Figure 3:**
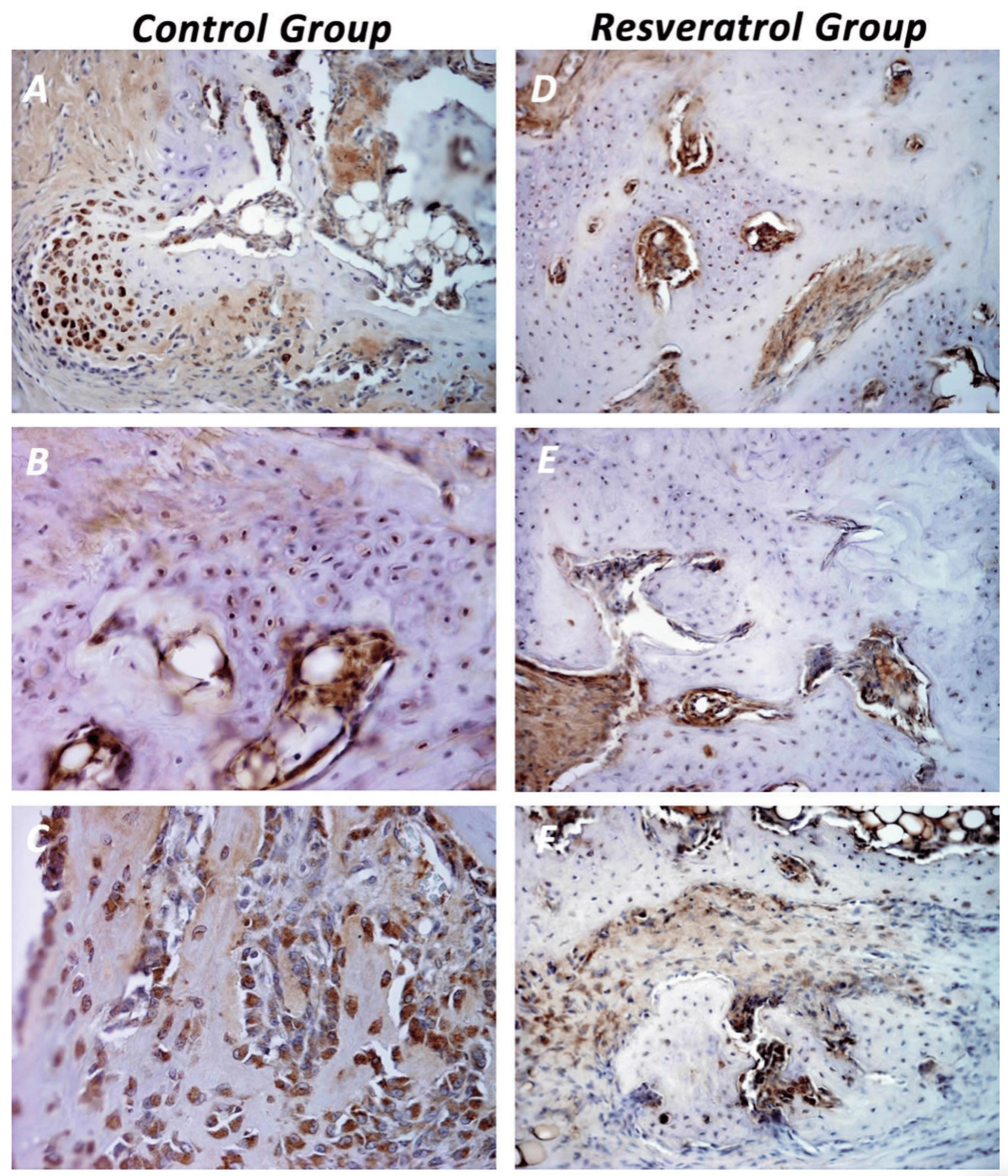
Immunohistochemical detection of 8-hydroxyguanine (8-OHdG) expression in fracture callus tissue. The representative images from the control group show (A) fibroblasts and chondrocytes with strong 8-OHdG expression (magnification 20×); (B) chondrocytes with both cytoplasmic and nuclear staining for 8-OHdG (magnification 40×); and (C) fibroblasts, chondrocytes, and osteoblasts displaying intense 8-OHdG immunoreactivity (magnification 40×). In contrast, sections from the resveratrol-treated group (D-F) demonstrate a mature, cell-rich callus predominantly composed of chondrocytes exhibiting low 8-OHdG expression (magnification 20×).

Sections were incubated with the primary antibody overnight at 4°C. The following day, they were treated with a biotinylated secondary antibody and an avidin-biotin-peroxidase complex (ABC kit), followed by 3,3′-diaminobenzidine (DAB) substrate to visualize positive staining. Counterstaining was performed with hematoxylin. Negative controls were included by omitting the primary antibody. Stained slides were evaluated under light microscopy. Immunoreactivity was assessed semi-quantitatively based on staining intensity and percentage of positive cells. The quantification was evaluated by staining intensity that was scored semi-quantitatively (0=none, 1=weak, 2=moderate, and 3=strong) across five random ×40 fields and confirmed using digital image analysis (Figure 3, panels A-F).

## Results

Two animals (one from each group) died suddenly during the study period from unknown causes. The remaining 18 animals (n=9 per group) completed the full protocol and were included in the analyses. All surviving rabbits tolerated the external fixation and distraction protocol without major complications. Pin tract infections were mild and resolved with standard wound care.

Posteroanterior radiographs taken immediately postoperatively confirmed appropriate fixator positioning and satisfactory alignment in all animals. Radiographs obtained on day 49 (one day before euthanasia) demonstrated progressive regenerate formation at the osteotomy site. No significant radiological differences in regenerating density or callus formation were observed between the control and experimental groups based on blinded scoring by two independent observers (p>0.05).

The baseline levels of 8-hydroxy-2’-deoxyguanosine (8‑OHdG) were comparable between groups. In the control group, 8‑OHdG increased significantly during the distraction phase, peaking at day 20 (p<0.01 vs. baseline) and remaining elevated through day 30 before partially normalizing by day 50. In the experimental group, resveratrol administration significantly attenuated the rise in 8‑OHdG during distraction (day 20 levels were 35% lower than controls, p<0.05) and normalized by day 50 (p<0.01 vs. controls) (Table 1). Moreover, the total antioxidant capacity (TAC) levels decreased during distraction in the control group (p<0.05 vs. baseline) and remained below baseline during consolidation. In the experimental group, TAC was significantly higher than controls at days 20, 30, and 50 (p<0.05 for all comparisons), suggesting enhanced systemic antioxidant defense with resveratrol (Table 1). Finally, the plasma glutathione (GSH) levels followed a similar trend to TAC. In controls, levels declined sharply at day 20 (p<0.01 vs. baseline) and only partially recovered by day 50. In the experimental group, GSH was significantly preserved at days 20 and 30 (p<0.05 vs. controls) and returned to baseline by day 50 (Table 1).

**Table 1:** Plasma oxidative marker levels (mean±SD) at different time points. *P<0.05 vs. control at the same time point. **P<0.05 vs. control at the same time point. Values in bold indicate significant change from baseline within group (p<0.05).

Markers	Group	Baseline (day 0)	Distraction (day 20)	Consolidation (day 30)	Endpoint (day 50)
8-hydroxy-2’-deoxyguanosine (8‑OHdG) (ng/mL) (0.156-0.210 ng/mL)	Control (n=9)	2.1±0.4	5.6±0.7	4.8±0.6	3.5±0.5
	Resveratrol (n=9)	2.0±0.3	3.6±0.5*	2.8±0.4	2.2±0.3**
Total antioxidant capacity (TAC) (mM Trolox eq.) (2.5-2.59 mM Trolox equivalent)	Control (n=9)	1.8±0.2	1.2±0.1	1.3±0.2	1.4±0.2
	Resveratrol (n=9)	1.9±0.2	1.6±0.2*	1.7±0.2	1.9±0.2**
Glutathione (GSH) (μmol/L) (9.4-10.4 μmol/L)	Control (n=9)	6.2±0.7	3.5±0.6	4.1±0.5	4.5±0.6
	Resveratrol (n=9)	6.3±0.6	5.1±0.5*	5.6±0.4	6.1±0.5**

At the 50th day, the immunohistochemical analysis of regenerated tissue revealed that the control group displayed intense nuclear staining of 8‑OHdG immunohistochemistry in fibroblasts (Figure 3, panel A), chondrocytes (Figure 3, panel B), and osteoblasts at the regenerate site (Figure 3, panel C), indicating persistent oxidative DNA damage. Contrariwise, the experimental animals showed markedly reduced 8‑OHdG staining intensity either in chondrocytes (Figure 3, panels D-F) and osteoblastic cells (Figure 3, panel D) (semiquantitative score: 1.2±0.3 vs. 2.6±0.4 in controls; p<0.01).

Finally, the distraction phase (days 10-20) was associated with a significant surge in oxidative stress in control animals, evidenced by increased plasma 8‑OHdG levels and decreased TAC and GSH levels. Resveratrol administration during this period significantly mitigated these changes, resulting in lower oxidative stress burden both systemically and in the regenerate tissue at the end of the study.

## Discussion

Distraction osteogenesis (DO) is a complex, staged biological process that relies on tightly regulated cellular, mechanical, and molecular interactions [10]. Although effective in treating critical-sized bone defects and limb length discrepancies, the prolonged treatment duration and risk of regenerate insufficiency remain significant barriers to optimal clinical outcomes [1]. The present study investigated the role of oxidative stress during DO, and it evaluated the impact of resveratrol administration, targeted specifically during the distraction phase, on systemic and local redox status in a rabbit femoral DO model.

Based on the literature reviewed, studies assessing antioxidant administration timed specifically to the distraction phase of DO in vivo appear to be limited or absent. The findings demonstrate that the distraction phase is accompanied by a distinct surge in oxidative stress, evidenced by elevated plasma and tissue levels of 8-hydroxy-2’-deoxyguanosine (8‑OHdG), alongside reductions in total antioxidant capacity (TAC) and glutathione (GSH). These results corroborate previous evidence indicating that reactive oxygen species (ROS) disrupt critical aspects of bone regeneration, including chondrocyte and osteoblast function, matrix mineralization, and the osteoimmune environment [2,3,11]. The high cellular turnover and mechanical strain inherent to the distraction phase may further potentiate oxidative damage, suggesting that this stage represents a critical therapeutic window for antioxidant intervention. Moreover, ROS have a wide range of downstream targets, integrating environmental stimuli and activating downstream molecular signal transduction cascades affecting neovascularization and regulating growth factor activity implicated in bone remodeling, such as fibroblast growth factors (FGFs), transforming growth factor beta (TGF-β), or inflammatory factors and Wnt/β-catenin pathway [12-17].

Resveratrol, a well-characterized polyphenol with potent antioxidant and osteogenic properties, was administered during this window with the aim of modulating oxidative stress and supporting bone regeneration. The experimental group exhibited significantly reduced systemic and local markers of oxidative damage during and after distraction, without adverse events or toxicity. Notably, immunohistochemical analysis of 8‑OHdG in regenerate tissue showed markedly decreased nuclear staining in osteoblasts and osteoclasts, suggesting direct cytoprotective effects of resveratrol at the site of active bone formation [18,19].

Although radiographic evaluation did not reveal statistically significant differences in regenerate density between groups, the biochemical and histological data provide strong evidence that resveratrol favorably modulated the redox environment during distraction. Given the established links between redox status, gene expression (e.g., RUNX2, COL10A1), and differentiation of osteogenic and chondrogenic lineages, it is plausible that resveratrol’s effect on mineralization would become more apparent at later consolidation time points or with complementary biomechanical testing [18,19].

Preclinical data outside of the DO context have shown that resveratrol enhances hypertrophic differentiation of chondrocytes and upregulates markers associated with endochondral ossification and matrix mineralization [5-7]. These mechanisms are particularly relevant to the latency and distraction phases of DO, during which chondrocyte-driven endochondral bone formation plays a vital role. Although our study did not directly assess chondrocyte mineralization, the attenuation of oxidative stress during distraction supports the hypothesis that resveratrol could protect and enhance the function of chondrocytes and osteoblasts alike.

Based on these findings, we propose a phase-specific antioxidant-based nutritional protocol for human patients undergoing distraction osteogenesis. During the distraction phase, the protocol includes resveratrol supplementation (100-250 mg/day), increased protein intake (1.5-2 g/kg body weight), and monitoring of redox status via the GSH/GSSG ratio at three key time points: at the onset, midpoint, and completion of distraction.

Furthermore, the impact of nutritional and metabolic factors on bone healing remains under-recognized in orthopedic practice. The dual role of diet - as a source of both protective (e.g., polyphenols, omega-3s) and deleterious (e.g., pro-oxidant micronutrient excess, such as iron) influences - suggests that individualized nutritional profiling could further enhance DO outcomes. Studies such as those by Nicolin et al. reinforce this perspective and align with the concept of an integrative osteometabolic approach [4].

However, our study has several limitations, including (a) the unclear direct impact of resveratrol on chondrocyte hypertrophy and mineralization, (b) the lack of evaluation of dose-response relationships and pharmacokinetics in the context of DO, and (c) the need to investigate the integration of antioxidant-based nutritional protocols into clinical care. Future studies should evaluate the long-term biomechanical properties of the regenerative process.

Additional limitations include the use of a single endpoint time for histological evaluation and the lack of pharmacokinetic assessment of systemic resveratrol levels. Although biochemical assays followed standardized protocols that do not require subjective interpretation, and the two unexpected deaths occurred equally between groups with no evidence of treatment-related toxicity, these factors nonetheless represent methodological constraints that can be addressed in future studies.

Finally, it should be acknowledged that precise radiographic quantification of regenerate length in small-animal distraction osteogenesis models is inherently challenging due to soft-tissue overlap, callus morphology, and the natural curvature of the femur. This limitation has been previously described in the rabbit DO literature and applies equally to both groups in the present study [20,21].

## Conclusions

This study provides novel evidence that oxidative stress increases significantly during the distraction phase of DO and that targeted antioxidant therapy with resveratrol effectively attenuates this surge at both systemic and local levels. These findings support the hypothesis that redox modulation during the critical window of distraction can improve the cellular environment necessary for successful regenerate formation. Although no differences in radiographic bone density were observed within the study’s time frame, the biochemical and histological data suggest that resveratrol exerts protective effects on bone-regenerating cells by reducing oxidative DNA damage and preserving endogenous antioxidant defenses. Ultimately, a personalized, phase-specific, redox-modulating therapeutic approach - combining pharmacologic agents like resveratrol with nutritional and metabolic profiling - may provide a novel paradigm for enhancing bone regeneration in distraction osteogenesis.
